# Model-based design of a pneumatic actuator for a dynamically reconfigurable socket for transtibial amputees

**DOI:** 10.3389/fbioe.2024.1459056

**Published:** 2024-12-23

**Authors:** Saeed Mollaee, Amir HajiRassouliha, David M. Budgett, Andrew J. Taberner, Poul M. F. Nielsen

**Affiliations:** ^1^ Auckland Bioengineering Institute, The University of Auckland, Auckland, New Zealand; ^2^ Department of Engineering Science and Biomedical Engineering, The University of Auckland, Auckland, New Zealand

**Keywords:** finite element method, pneumatic soft actuator array, adjustable prosthetic socket, image registration algorithm, hyperelastic modelling, computer vision, reconfigurable socket

## Abstract

In this work, a cost-effective, scalable pneumatic silicone actuator array is introduced, designed to dynamically conform to the user’s skin and thereby alleviate localised pressure within a prosthetic socket. The appropriate constitutive models for developing a finite element representation of these actuators are systematically identified, parametrised, and validated. Employing this computational framework, the surface deformation fields induced by 270 variations in soft actuator array design parameters under realistic load conditions are examined, achieving predictive accuracies within 70 µm. The results elucidate how individual design factors influence surface deformation and, consequently, pressure distribution. A novel speckle imaging technique is employed to address the complex non-linear deformations, enabling surface displacement measurements with an accuracy of approximately 40 µm. These measurements confirm that the Ogden N3 model can predict actuator deformation with an accuracy of 16%. These findings elucidate the relationships among actuator geometry, material behaviour, and surface deformation. Although demonstrated in a dynamically reconfigurable socket for transtibial amputees, these insights are readily transferable to other robotics applications that require soft, deformable, load-bearing interfaces. This validated modelling strategy and imaging technique provide a foundation for optimising soft actuator arrays, ultimately improving user comfort and enhancing the functionality of future prosthetic and robotic devices.

## Introduction

The global incidence of lower limb amputation is escalating, primarily from diabetes and its consequent 8- to 24-fold increase in rates of amputation (Fosse et al., 2009). The World Health Organisation reported 415 million diabetes cases in 2015, with predicted growth to 642 million in 2040 and a corresponding increase in amputations (W. H. Organization and USAID). In 2005 it was estimated that in United States alone about 1.6 million people suffered lower limb amputation, projected to increase to 3.6 million by 2050 (Ziegler-Graham et al., 2008).

**FIGURE 1:**
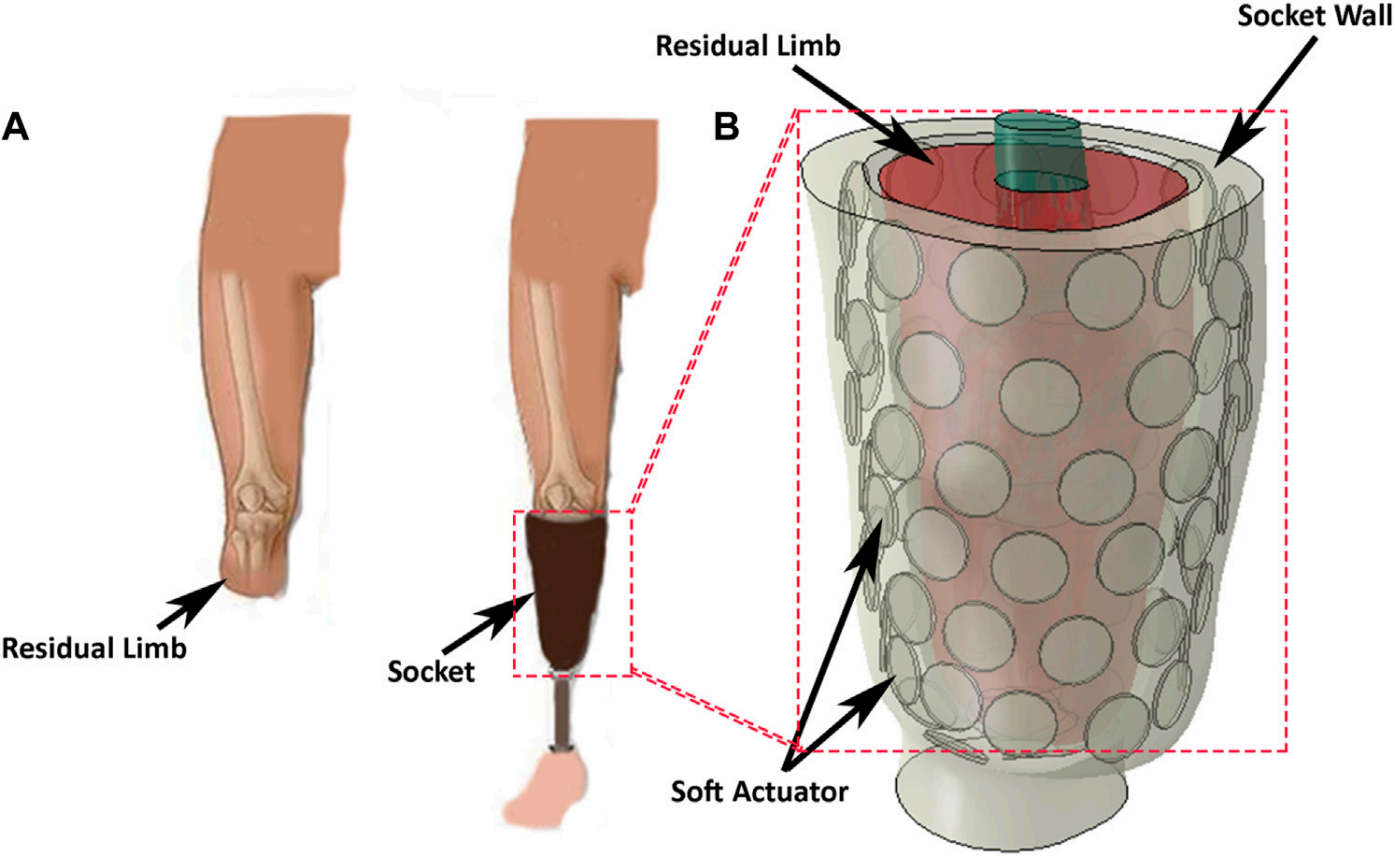
**(A)** A transtibial left-amputee, wearing a socket and prosthesis, and with the prosthesis removed. **(B)** Concept of a reconfigurable socket incorporating a soft-actuator array to manage the interface between the prosthesis and the skin surface of the stump.

Limb amputations cause severe physical disabilities which affect the amputee’s quality of life. One of the most common treatments to restore lost functionality following lower-limb amputation is to use a prosthetic leg that attaches to the residual limb *via* a socket (Figure 1A). In transtibial amputees, the body load must be supported by the surface of the residual limb (stump), resulting in high stresses in the residual limb soft tissues (Sanders and Daly, 1993). But soft tissues are typically not well-suited for carrying such high stresses.

Two socket designs are commonly used to minimise tissue damage and alleviate pain. The first of these is the patella tendon bearing (PTB) socket design, which applies loads to regions of the residual limb known as “pressure tolerant” areas. The second is the total surface bearing (TSB) socket design which distributes the load almost uniformly over the residual limb surface. Both designs have shortcomings, the most common being fit and comfort (Baars et al., 2018), (Sherman, 1999), (Pezzin et al., 2004).

Socket comfort depends principally on the pressure distribution at the interface between the socket and the residual limb (Mollaee et al., 2024). In one study of approximately 600 amputees, more than 60% were dissatisfied with their prostheses (Sinha et al., 2011) mainly because of discomfort resulting from poor socket fit. Often poor fit arises due to residual limb volume fluctuation (Gailey et al., 2010), (Paternò et al., 2018), which can result in pressure concentrations at the socket/stump interface. Regions of the stump that are overloaded for long durations can experience poor blood circulation and severe damage, such as vascular occlusions (Meulenbelt et al., 2009), skin irritation, pressure ulcers, and other dermatological problems (Lyon et al., 2000). Thus, controlling the pressure distribution at the socket/stump interface may reduce discomfort and skin-related problems (Mirjavadi et al., 2021).

Over the past few decades, work on transtibial socket design has concentrated on enhancing the socket fit and controlling the pressure distribution. Previously, sockets had a generic residual limb shape, with a tight corset to partially off-load the stump, or used a hydrostatic technique to provide uniform pressure distribution over the stump (Sewell et al., 2000). Some sockets used a combination of these techniques to improve socket fit (Pirouzi et al., 2014), (Stevens et al., 2019), adjusting stiffness at particular regions (Sengeh and Herr, 2013a) to control the pressure distribution or including movable sections to adjust the socket size and compensate for stump volume fluctuation (Weathersby et al., 2022).

Kahle et al. (2020) stated that socket design significantly impacts both gait stability and comfort in prosthetic users, with discomfort frequently reported in commonly used designs, such as the ischial ramus containment socket. Their study observed no significant differences in comfort or skeletal motion among ischial ramus containment, dynamic, and sub-ischial sockets, suggesting that alternative designs could provide comparable support without compromising comfort. Åström and Stenström (2004) mentioned that the polyurethane socket concept significantly enhanced comfort and physical capacity in transtibial amputees compared to conventional suspension systems. Despite these improvements in comfort, gait registration did not provide useful insights into amputees’ satisfaction or socket comfort. Hsu et al. (2018) illustrated that the design of the prosthetic socket is a key factor influencing the comfort of amputees, as improper fitting can lead to localized pressure points and discomfort. They further emphasized that advancements in socket design, such as improved pressure relief and better interface stress distribution, can significantly enhance comfort and overall user satisfaction.

In recent years, the development of ‘soft robotics’ (El-Atab et al., 2020) has found many applications from manufacturing to healthcare (Rosso et al., 2005; Morales et al., 2014). In prosthetic design, soft pneumatic sensorised liners have been used inside transtibial sockets to accommodate volume change in residual limbs and allow pressure control on the residual limb (Carrigan et al., 2016) which are mainly made of hyperelastic materials, especially elastomers like rubber, silicone, and some types of polyurethane, are widely studied for their behavior under different loading conditions (Mollaee et al., 2023). Hyperelastic modeling of the skin, using a three-parameter hyperelastic model, is crucial for optimizing prosthetic socket designs by accurately simulating the skin’s nonlinear deformation and ensuring a better fit to reduce discomfort and pressure points. Recently, several researchers have focused on modeling hyperelastic structures for use in biomedical applications, including prosthetic socket design (Afshari et al., 2022; Mirjavadi et al., 2020; Forsat, 2020). Others Paterno et al. (2022) have added an actuator between two fibre layers to create an inner flexible socket for transfemoral amputees.

High costs and limited functionality in existing lower-limb prosthetic solutions have created a need for advanced, multifunctional devices. To address this, new prosthetic socket designs should integrate soft actuators that facilitate real-time pressure sensing, enhancing user comfort and fit. Additionally, incorporating myoelectric sensors, as demonstrated in recent gait phase recognition studies, could significantly improve the controllability and adaptability of these devices, enabling smoother, more natural movements for users (Tigrini et al., 2024).

Despite these advances there remain several shortcomings with prosthetic sockets (Paternò et al., 2018), (Mak et al., 2001). Principal among these is poor control of the displacement of soft actuators in prosthetic sockets, and designs that are complicated to manufacture (Weathersby et al., 2022; Carrigan et al., 2016; Paterno et al., 2022). Furthermore, no study has thoroughly characterised the behaviour of a soft actuator array under realistic loading conditions and provided an easily-manufactured, cost-efficient, and simple design for a soft actuator array (Mollaee et al., 2023). Consequently, no device has yet been constructed that enables the user to redistribute the pressure over the whole residual limb, nor control socket volume fluctuation over stump.

Here, we present the design of a pneumatic actuator array suitable for use in a dynamically reconfigurable socket (Figure 1B). The array comprises multiple independent pneumatic actuators that can be reconfigured to control the internal shape of the socket and/or the pressure distribution at the socket/stump interface. This approach provides control over the pressure distribution and volume adjustment over the stump and may alleviate discomfort by temporarily or permanently removing the load from sensitive areas.

We first detail the design and construction of a soft actuator array constructed from a silicone elastomer. Next, we model the array with a suite of finite element (FE) models, using a variety of constitutive relations for the elastomeric material. The predictions of the models are validated by using a force/torque transducer to indent the surface of a prototype soft actuator, while the shape and deformation of the soft actuator array surface is profiled at high-resolution. We thus identify the constitutive relation and material parameters that best predict actuator deformation. Next, we use the model to study the effect of design parameters on the surface deformation and surface pressure distribution of an array of actuators. Finally, we discuss how each design parameter affects the soft actuator surface pattern and surface displacement.

## Methods

### Design requirements

During normal activity, the load experienced by the stump greatly exceeds the static load imposed by the user’s weight. Several studies have investigated the pressure at different regions of the residual limb during various activities, reporting the shear and normal stress, or simply the pressure (Ko et al., 2018; Sanders et al., 1998; Dou et al., 2006; Rajtukova et al., 2014; Sengeh and Herr, 2013b; Swanson et al., 2018). Based on these data, it is, reasonable to assume that the maximum normal stress and maximum shear stress developed at the stump-socket interface is limited to approximately 200 kPa and 10 kPa, respectively.

We propose that the inner surface of a socket be assembled from an array of soft actuators to allow control over the surface shape and/or the pressure distribution applied to the stump (Figure 1A). To explore the feasibility of this proposition, we constructed a linear array of four pneumatically driven disc-shaped ‘voids’ embedded in a silicone elastomer. Platinum-catalysed silicones were selected for the actuators because they are flexible and are able to be subjected to repeatable strains of over 150% without damage (Smooth On Ecoflex 00-50).

**FIGURE 2:**
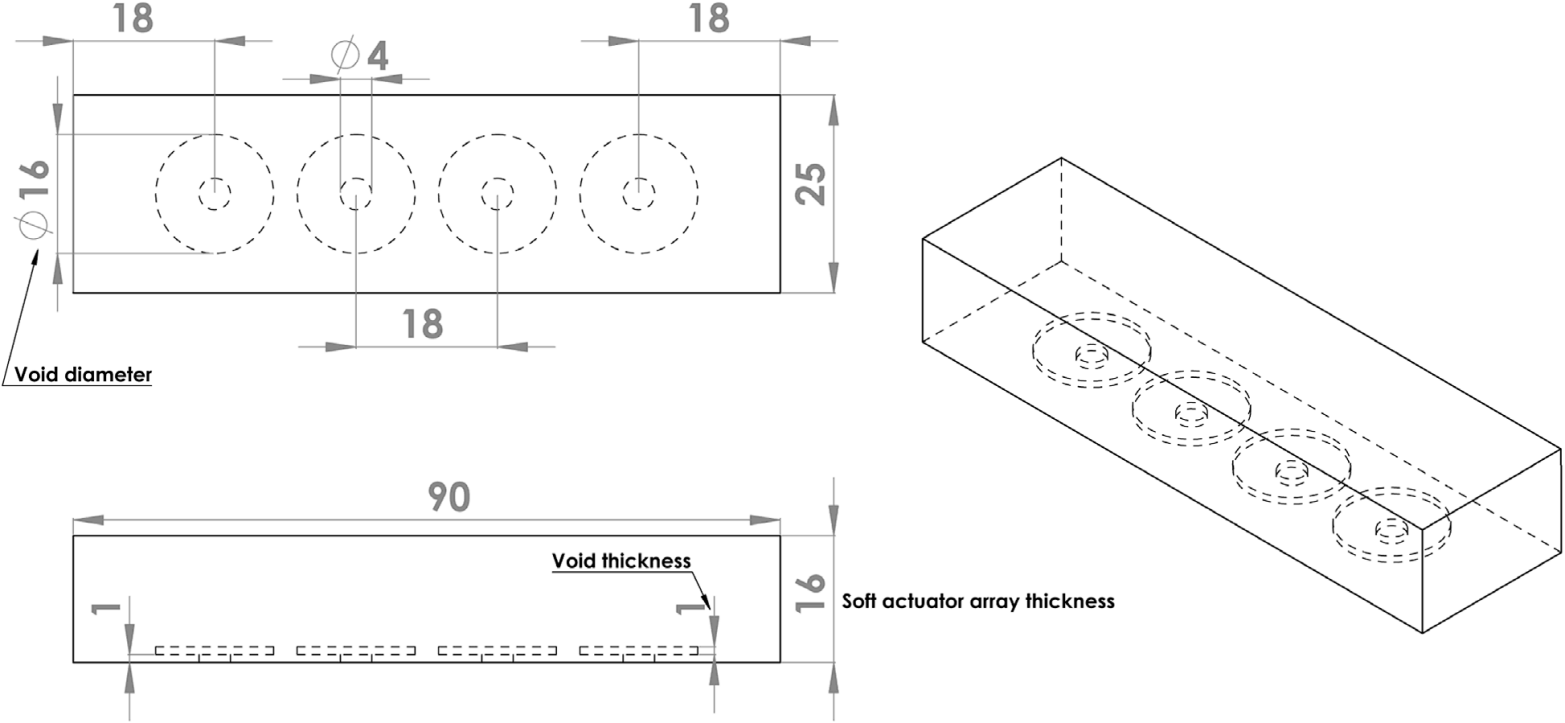
Soft actuator array design. All dimensions are in mm.

A proof-of-concept array comprised four independently controlled actuators spaced along the length of a rectangular block of silicone (Figure 2). Each actuator was operated by inflating the void with air at controlled pressure, thereby extending the silicone, predominantly in the axial direction of the void. The bottom surface of the actuator was fixed to a stiff base layer of acrylic (not shown). The soft actuator array was 90 mm long, 25 mm wide, and 16 mm thick, dimensions suitable for embedding in the proposed socket. The void diameter was set to 16 mm, with voids spaced 18 mm apart, resulting in an equal distance between the void centres.

### Soft actuator construction

**FIGURE 3:**
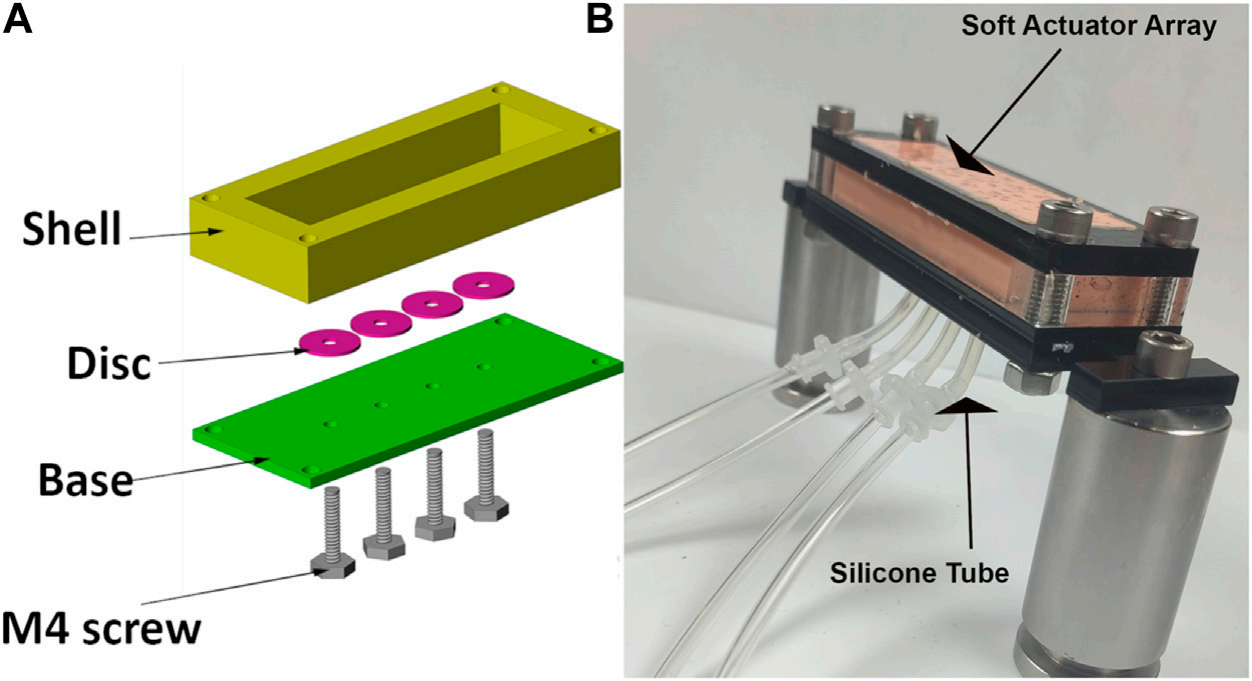
**(A)** mould components to construct the soft actuator arrays. Disc used to form the void and held in location during curing with M4 screw. **(B)** Photograph of assembled soft actuator arrays and silicone tubes connected to each void.

The soft actuator array was cast in an acrylic mould which comprised a shell producing the desired thickness for the soft actuator, four discs to form voids inside the soft actuator base, and four screws for holding the voids at the desired distance from the base. Two-part silicone (Ecoflex 00-50) was mixed and degassed in a vacuum chamber to eliminate entrapped air and poured into the mould. Following curing at room temperature for 12 h, the discs forming the voids were removed through the screw holes, which were later used for inflation. Silicone tubes were adhered to the holes on the base and used to pressurise each void individually. A black speckle pattern was airbrushed on the actuator surface to generate a wide-spatial-density random pattern for optical tracking using a stereo image reconstruction algorithm (Figure 3).

### Measuring soft-actuator array performance

Actuators were driven by applying, independently, an air pressure to each void, using four electronic pressure regulators (ITV1000, SMC Corporation, Japan). Each regulator was controlled through an analogue input/output device (National Instruments myRIO-1900) from LabVIEW 2019 (NI). The regulators were connected to the actuators using silicone tubes with internal and external diameters of 2 mm and 4 mm respectively. The actuators were pre-inflated to a pressure of 60 kPa.

**FIGURE 4:**
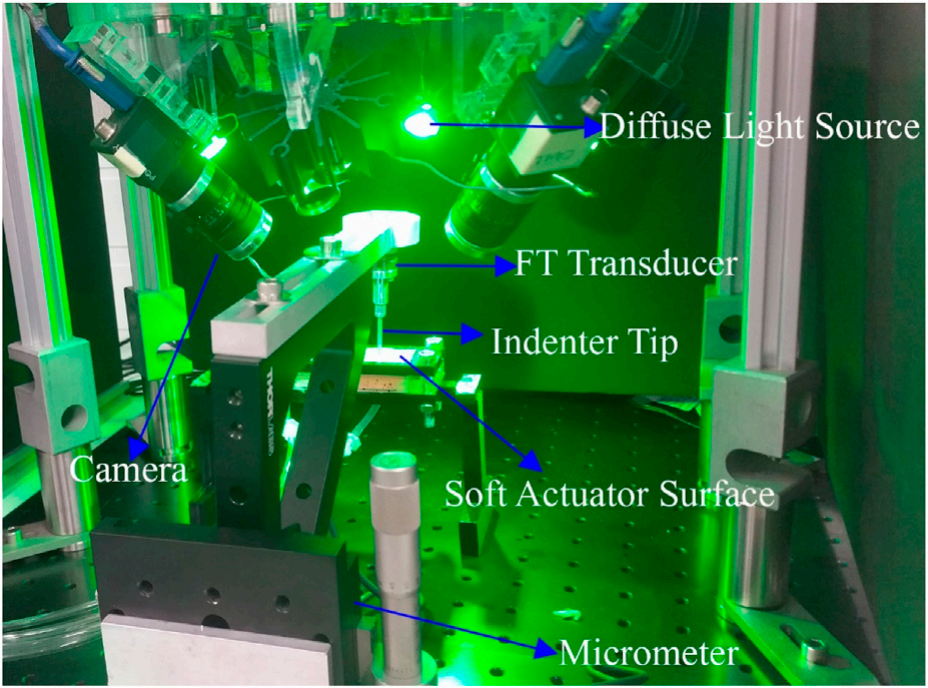
The indentation experiment setup. Micrometre and four camera stereoscope performing indentation experiment on an Ecoflex 0050 soft actuator. Force-torque transducer records the force data during the indentation, and cameras take a picture from four different views at each step.

Actuator force/torque production and deformation was measured using a mechanical testing apparatus. A six-axis force-torque transducer (nano 17, ATI Industrial Automation) with an attached cylindrical tip (2.66 mm diameter) was manually advanced against the actuator array surface by a micrometer. The 3D reaction force vector was measured while the micrometer was advanced by a displacement measured to 10 µm resolution (Figure 4). The deformation of the actuator surface was measured across a rectangular area of 15 mm × 25 mm centred on the location of the indenter tip, using a custom stereoscopic imaging system. The indentation started at 0 mm and ended at 2.5 mm, after 25 increments of 100 µm.

Surface deformation was measured using a stereoscopic system consisting of four machine-vision cameras (Flea3 FL3-U3-13Y3M, Teledyne FLIR LCC, United States), each equipped with 6 mm lenses (Fujinon Lens DF6HA-1B, Fujifilm Corporation, Japan). To reduce specular reflections, circular polarisers (PL-CIR S 27 mm/0.75, Hoya Corporation, Japan) were attached to each lens. Cameras were mounted at 45° on acrylic blocks at the four corners of a rectangular aluminium optical breadboard, with sides approximately 225 mm and 150 mm (Figure 4). Software-triggered image acquisition was performed in at a rate of one capture per indentation increment. Surface illumination was provided by four green (560 nm) light-emitting diodes (LEDs), mounted between the cameras. Identification of the cameras’ intrinsic and extrinsic parameters was achieved using a multi-camera calibration technique, which automatically calculated parameters from sets of calibration images of a checkerboard pattern (Haji Rassouliha, 2017).

### Surface profiling

The calibrated stereoscope system was used to reconstruct the 3D geometry by integrating the overlapping views of at least two cameras using the method of HajiRassouliha et al. (2019) for surface profiling. We applied biquadratic polynomial transforms into three views to align with the view from a fourth camera, known as the reference camera.

**FIGURE 5:**
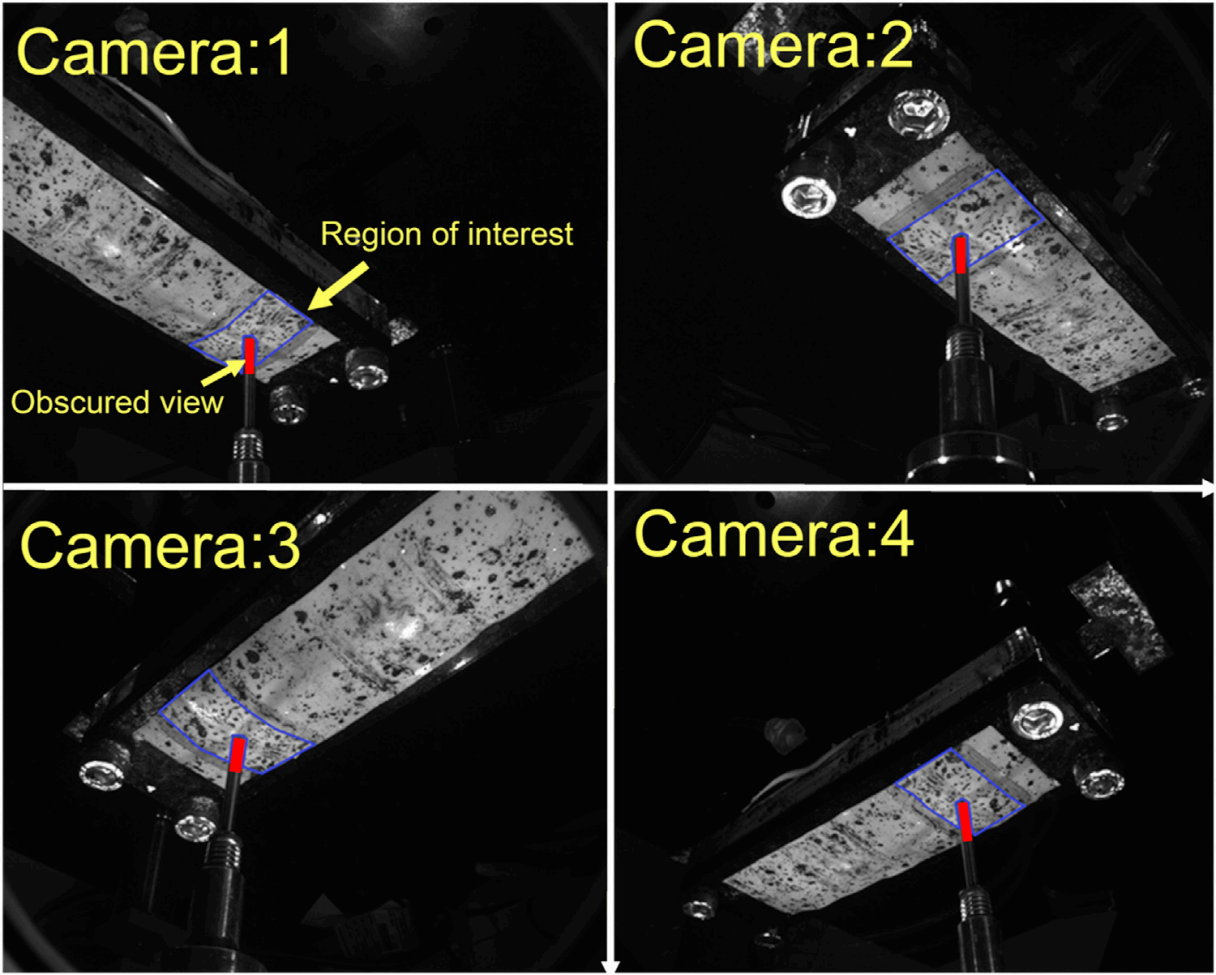
View from four different cameras. Region of interest for 3D reconstruction coloured by blue-bordered area. View obscured by indenter tip is coloured by red.

At each pair view (the overlapping view of two cameras), approximately 90 distinct points were matched as an initial guess to estimate the surface shape and deformation. To generate the new position of the features at each indentation step, we used the subpixel image registration algorithm of HajiRassouliha et al. (2017) with a 64 pixel × 64 pixel window. This algorithm generated a point cloud corresponding to the surface shape at each indentation step, including the new coordinates of the tracked points in 3D (HajiRassouliha et al., 2019; HajiRassouliha et al., 2013) When imaging the soft actuator under the indentation, some areas of the surface were obscured due to occlusion by the indenter tip. To address this issue, the coordinates from non-obscured cameras were used to reconstruct the surface and compensate for the missing data in the reference camera view (Figure 5).

### Finite element models

The behaviour of the soft actuators can be predicted by the FE method after identifying a hyperelastic constitutive relation suitable for describing silicone rubber. Previous studies have typically a variety pf constitutive relations to characterise the mechanical behaviour of silicone materials. Researchers have previously used Ogden N3 (Elsayed et al., 2014; Choi et al., 2017), Yeoh (Sarkar et al., 2019), and Arruda-Boyce (Shivapooja et al., 2015) hyperelastic relations to model the behaviour of Ecoflex 0050. The lack of consensus in the above literature led us to perform our own analysis to identify an appropriate hyperelastic relation for Ecoflex 0050.

We employed commercially-available FE analysis software (ABAQUS Inc., Dassault Systems Corp) to perform finite element simulations. We developed and evaluated finite element models using six different hyperelastic constitutive relations to find a suitable model for Ecoflex 0050.

• Reduced Polynomial Order 1 (Neo-Hookean)
• Reduced Polynomial Order 2 (Reduced Polynomial N2)
• Reduced Polynomial Order 3 (Yeoh)
• Arruda-Boyce
• Ogden Order 1 (Ogden N1)
• Ogden Order 3 (Ogden N3)

Uniaxial stress-strain data were acquired from dogbone-shaped samples of Ecoflex 0050 (as recommended in ASTM standard D412) using an electro-mechanical universal testing machine (Instron 5567). ABAQUS’s inbuilt optimisation package was then used to estimate the best-fit coefficients for each hyperelastic constitutive model (“Fitting of hyperelastic and hyperfoam), assuming the material to be isotropic and incompressible (Martins et al., 2006; Asadi Khanouki et al., 2019). (Table 1) shows the best-fit coefficients optimised by ABAQUS for each constitutive relation.

**TABLE 1:** Constitutive relation coefficients, fitted by ABAQUS to uniaxial experimental measurement.

Model	Model Equation	Coefficient	Model	Model Equation	Coefficient
**Neo-**Hookean	He et al. (2021) $W=C_{10}\left(I_{1}-3\right)$	C_10_ = 22.4 kPa	**Yeoh**	$W=\sum_{i=1}^{N=3}C_{i0}{\left(I_{1}-3\right)}^{i}$ (He et al., 2021)	C_10_ = 21.9 kPa C_20_ = 0.0690 kPa C_30_ = 0.0167 kPa
Reduced Polynomial N2	Dias et al. (2014) $W=\sum_{i=1}^{N=2}C_{i0}{\left(I_{1}-3\right)}^{i}$	C_10_ = 20.2 kPa C_20_ = 0.0575 kPa	Ogden N1	$W=\sum_{i=1}^{N=1}\frac{2\mu_{i}}{\alpha_{i}^{2}}\;\left(\lambda_{1}^{{-\alpha }_{i}}+\lambda_{2}^{{-\alpha }_{i}}{+\lambda }_{3}^{{-\alpha }_{i}}\right)$ (He et al., 2021)	μ = 39.5 MPa α = 2.41
**Arruda-Boyce**	Arruda and Boyce (1993) $W=\mu \sum_{i=1}^{N}\frac{C_{i}}{\lambda_{m}^{2i-1}}\;\left({\bar{I}}_{1}^{i}-3^{i}\right)$ $C_{1}=\frac{1}{2}$ *,* $C_{2}=\frac{1}{20}$ *,* $C_{3}=\frac{11}{1050}$ $C_{4}=\frac{19}{7000}$ *,* $C_{5}=\frac{519}{673750}$	λ_m_ = 2.42 µ = 36.1 kPa	Ogden N3	$W=\sum_{i=1}^{N=3}\frac{2\mu_{i}}{\alpha_{i}^{2}}\;\left(\lambda_{1}^{{-\alpha }_{i}}+\lambda_{2}^{{-\alpha }_{i}}{+\lambda }_{3}^{{-\alpha }_{i}}\right)$ (He et al., 2021)	μ_1_ = −37.1 kPa μ_2_ = 23.1 kPa μ_3_ = 70.2 kPa α_1_ = 1.63 α_2_ = 3.36 α_3_ = −2.92

Having identified the optimised coefficients for constitutive models for Ecoflex 0050, we then validated a FE model of our actuators. Model results were compared to data recorded during an indentation test conducted on our prototype actuators. Literature on the normal and shear stress on the stump indicate maximum normal and shear stresses of 200 kPa and 10 kPa, respectively. We thus applied these stresses in our FEM studies of soft actuator arrays. The array was constrained in all directions except the surface to which the external load was applied (Figure 3).

During indentation, we controlled the indentation depth and recorded the force produced at each step. The experimentally recorded forces were used as 3D boundary forces to indent the soft actuator surface in the FE model. The displacement of the indented area was exported from the FE model at each indentation step for each hyperelastic model and compared with the experimental data. We also compared the measured displacement of points neighbouring the indenter tip with those predicted by the FE model.

### Design parameter study

In the second part of the study, we investigated the behaviour of the soft actuator array under various loading conditions, as design parameters (e.g., thickness, void size, input pressure) were varied. We investigated different ranges of design parameters to evaluate their effect.

• Input Pressure (*P*) above atmosphere: 150 kPa, 175 kPa, 200 kPa, 225 kPa, 250 kPa
• Void Diameter (*VD*): 8 mm, 12 mm, 16 mm
• Void Thickness (*VT*): 1 mm, 2 mm
• Soft Actuator Thickness (*T*): 8 mm, 12 mm, 16 mm

To investigate the consequences of different combinations of the design parameters, the input pressures were varied up to the maximum reported pressure (i.e. 200 kPa). Other geometric properties were selected such that the soft actuator surface reached about 5 mm, which is a comfortable range adjustment for amputees (McLean et al., 2019) and also enables the dynamically reconfigurable socket to accommodate −11%–7% of residual limb volume fluctuation (Board et al., 2001). Combinations of the design parameters were studied under normal stress, shear stress, and a combination of both, yielding a total of 270 models. All models used the Ogden N3 hyperelastic constitutive relation.

The range of selected design parameters also enable us to investigate each parameter’s effect on the surface deformation, and demonstrate the combination that can produce concave and convex surface shapes, and uneven and smooth surface shapes. Based on the stresses reported in the literature (summarised in Table 2), the normal external stress and shear stress applied on the surface of the soft actuator array were set at 200 kPa and 10 kPa, respectively.

**TABLE 2:** Maximum stump-socket interface stresses and pressures for lower limb amputees.

Study	Normal stress (kPa)	Shear stress (kPa)	Pressure (kPa)
Ko et al. (2018)	159.3	4.2	—
Sanders et al. (1998)	78.8	10.1	—
Dou et al. (2006)	—	—	215.8
Rajtukova et al. (2014)	—	—	81
Sengeh and Herr (2013b)	—	—	58
Swanson et al. (2018)	—	—	60

## Results and discussion

### Constitutive relation evaluation

**FIGURE 6:**
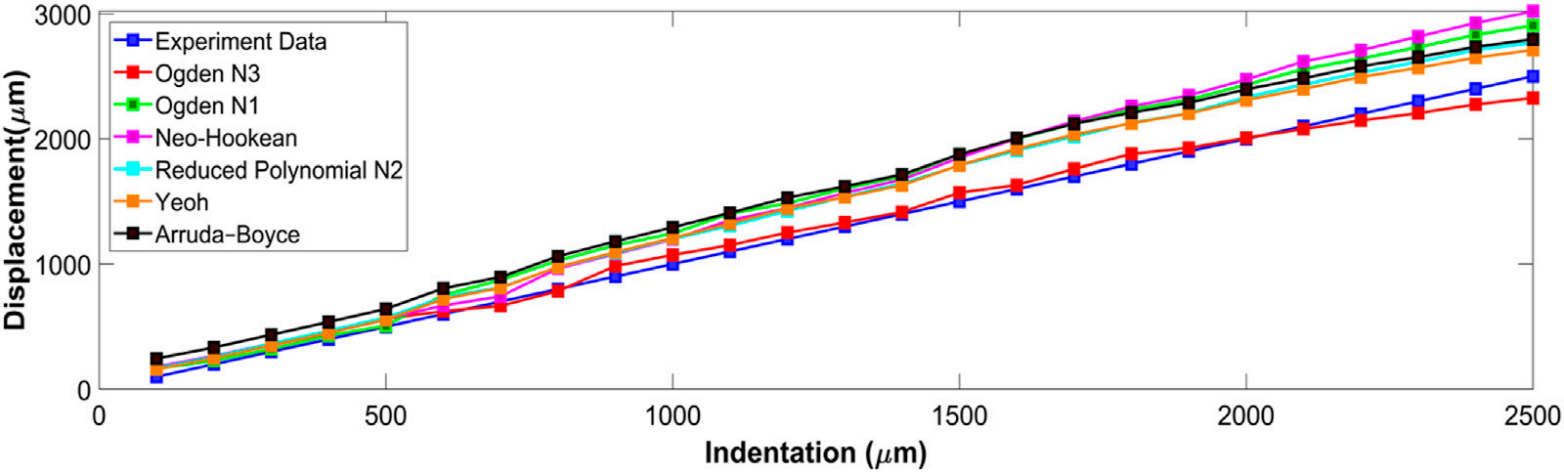
Predicted indentation for all constitutive relations compared to experimental data.

Six hyperelastic constitutive relations were investigated by comparing FE model predictions with experimental measurements at each indentation step (Figure 6). displays the mean displacement of the indentation area, and compares the experiment tip indentation depth to that predicted by the FE model using each constitutive relation. The predictions of Ogden N3 were closest to the experimentally measured displacements with a maximum error of 173 μm at full indentation. Neo-Hookean had the largest discrepancy with indentation depth, with a maximum discrepancy of 527 μm at full indentation (Figure 6).

The RMS error for each constitutive relation is listed in Table 3. Ogden N3 displayed the best performance with 68 µm RMS error, with the next best constitutive relations being Yeoh and Reduced Polynomial N2 with 223 μm and 241 µm RMS error, respectively.

**TABLE 3:** The table shows the RMS error of the indentation region for all constitutive relations. The Ogden N3 relation had the lowest RMS error.

Constitutive relation	RMS error (µm)
Ogden N3	68
Ogden N1	320
Neo-Hookean	338
Reduced Polynomial N2	241
Yeoh	230
Arruda–Boyce	310

Fitting the surface of the dense point cloud, corresponding to the soft actuator surface, generated an RMS error of 42 µm. The location of the indenter tip in the stereoscopic images caused an approximately 3 mm × 3 mm area of missing points surrounding and beneath the indenter tip. FE models were fitted to the point clouds to quantify the discrepancy between the predictions and experimental displacement.

**FIGURE 7:**
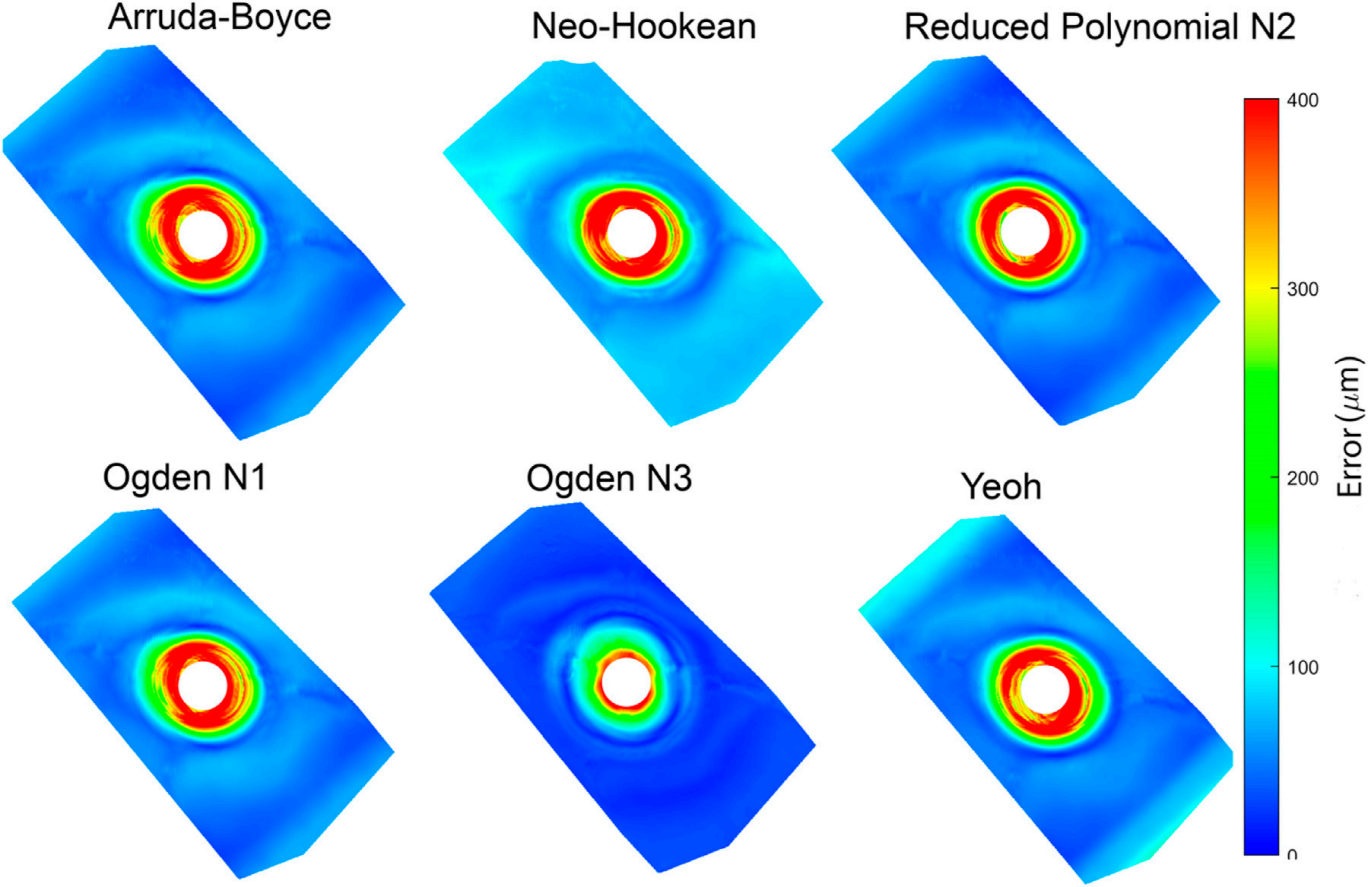
The average error between the stereo reconstruction and six different FEM predictions.

The absolute displacement difference between the FE models and point cloud measured at each step, and the average of the discrepancy for all steps, were calculated and are depicted in (Figure 7). The error was highest (∼400 µm) close to the point of indentation, but gradually decreased towards the edges (<150 µm). Of the constitutive relations considered, the Ogden N3 yielded the closest predictions to measured deformations, while the Neo-Hookean relation had the highest error. The Ogden N3 constitutive relation was thus selected for all subsequent model analyses.

### Design parameter study

**FIGURE 8:**
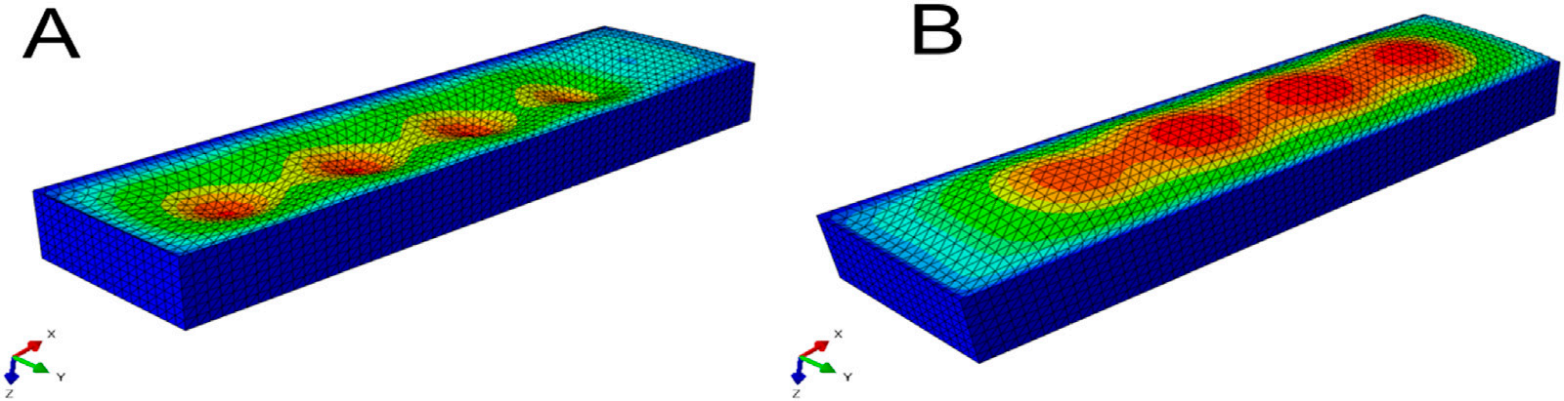
Picture **(A)** shows the soft actuator in the negative-deformation condition, and picture **(B)** displays the soft actuator in the positive-deformation condition.

Next, we used the model to investigate the effect on surface shape of four main design parameters: void diameter (*VD*); void thickness (*VT*); soft actuator thickness (*T*); and input pressure (*P*) (Figure 2). The resulting shape of the soft actuators for the various combinations of design parameters belong to two main groups. The first group is a *negative-deformation* surface shape (Figure 8A) in which the external loading overcame the load applied from the soft actuator, and resulted in surface concavity. The second group is exhibited *positive-deformation* (Figure 8B) where the soft actuator surface generated sufficient force to overcome the external stresses applied to the surface, and producing a convex shape.

**FIGURE 9:**
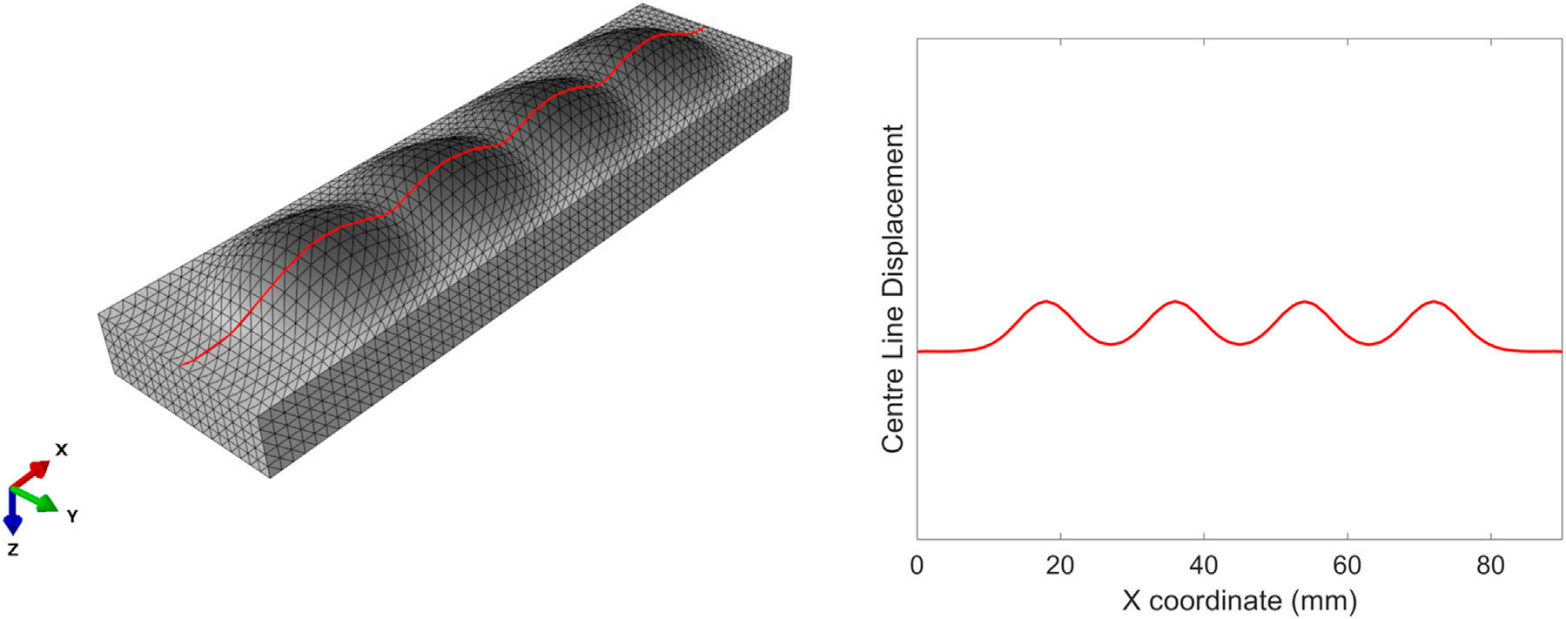
Centre line on the left (in red) along which the surface deformation of the soft actuators is extracted (right).

To evaluate the effect of each design parameter on the soft actuator surface shape under loading, it is helpful to visualise and compare the surface data. Due to the complexity of visualising 270 full 3D surface profiles, data were extracted along the *y*-centre line (*x* = 0) of the soft actuator (Figure 9).

**FIGURE 10:**
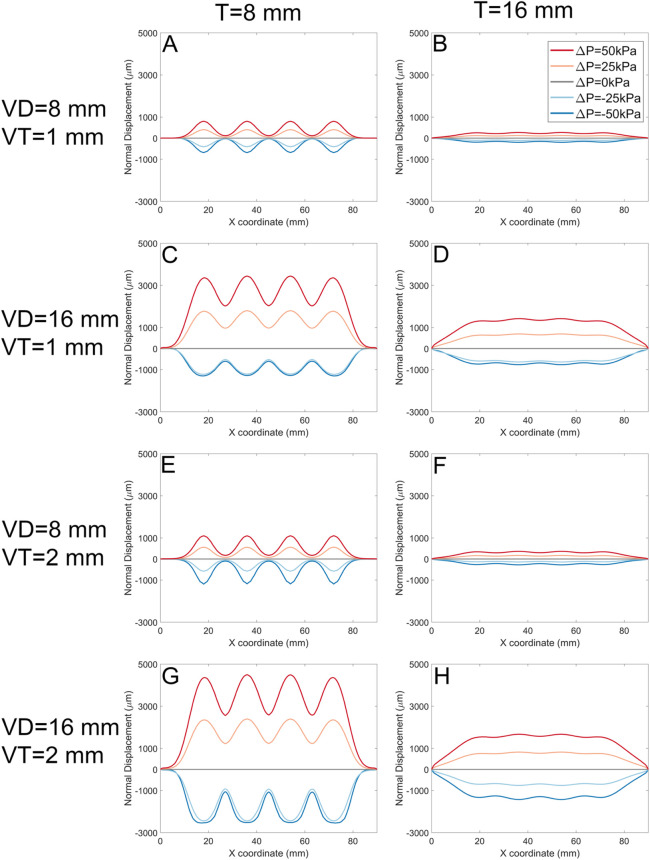
The normal displacement for models with 1 mm, and 2 mm void thickness (VT). Each panel represents data for a specific soft actuator thickness (T), void diameters (VD), and VT. Across panels, lines with the same colour represent the same soft actuator pressure. The first row of the panels **(A, B)** belongs to the data with the VD 8 mm, VT 1 mm, and T 8 mm, and 16 mm, respectively. The second row **(C, D)** demonstrates the results for the VD 16 mm, VT 1 mm, and T 8 mm, and 16 mm, respectively. The third row **(E, F)** demonstrates results for analysis with the VD 8 mm, VT 2 mm, and T 8 mm, and 16 mm, respectively. The fourth row **(G, H)** demonstrates FE results for the VD 16 mm, VT 2 mm, and T 8 mm, and 16 mm, respectively.

By extracting these data, we can plot the various types of soft actuator array surface deformation that can be achieved by manipulating the design parameters (Figure 10). Each panel in Figure 10 shows the vertical displacement for the soft actuator array with a specific void thickness, void diameter, and actuator thickness, at five pressure differences between the void pressure and the external stress.

In each panel, the pressure difference can change the surface deformation from negative to positive. For positive *ΔP*, the surface displacement magnitude is approximately proportional to *ΔP* regardless of other design parameters. In some cases (Panels A, H, where *VD* = *T*) the deformation in response to -*ΔP* is approximately the inverse of that to *ΔP.* However, in other cases (Panels C, E, G) negative pressure difference leads to ‘collapse’ of the actuator void towards its minimum possible thickness as the top surface of the void contacts the bottom surface.

The soft actuator array thickness *T* (left column vs. right column) has an inverse effect on the magnitude of the surface displacement, and results in a smoother surface. On the other hand, larger *T* reduces local control over surface deformation with pressure. The overall surface displacement increases with increasing void diameter (e.g., Figure 10, A cf C, E cf G). Smaller void diameters cause the mid points between actuators to remain constrained to near-zero displacement; larger void diameters *VD* allow the entire actuator surface to offset in the positive direction.

Increasing the soft actuator array thickness and increasing the void diameter simultaneously (Figure 10A, and D) gradually increases the surface displacement magnitude and provides a more uniform surface deformation. Increasing the void thickness (*VT*) allowed slightly larger deformation (Figure 10, A cf E, C cf G).

**FIGURE 11:**
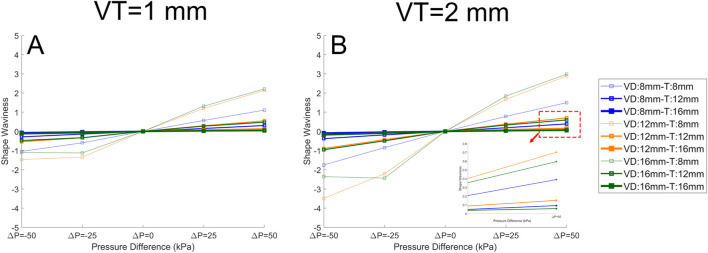
The quantified surface shape for the soft actuator arrays when the VT (void thickness) is 1 mm **(A)**, and the VT is 2 mm **(B)**. The pressure difference is plotted against the average peak-trough percentage for each model divided by the average peak in all models (shape waviness). It is clear that pressure difference has a significant effect on the shape waviness, and on the other hand, increasing thickness reduces the shape waviness. In the legend the “VD” stands for void diameter and “T” stands for soft actuator array thickness. The lines with the same color have the same “VD”, and the lines with the same thickness have the same “T”.

These data can be further analysed by quantifying the *shape* of the surface (Figure 11). Here, we define the ‘shape waviness’ as the average of peak-trough amplitude for each model divided by the average of the peaks’ magnitude across all models; positive shape waviness indicates convex surface deformation (Figure 8B) and negative shape waviness indicates concave surface deformation (Figure 8A):

$$∆AveragePeak=\left(\frac{\sum_{1}^{4}PeakValue}{4}\right)-\left(\frac{\sum_{1}^{3}TroughValue}{3}\right)\;Average_{AllPeaks}=\left(\frac{\sum_{1}^{i}PeakValue}{i}\right)\;Shape\;waviness=\frac{∆AveragePeak}{Average_{AllPeaks}}$$

Shape waviness increases with void thickness *VT* (Figure 11A CF Figure 11B), and decreases with array thickness *T*. Under a negative pressure difference, any increase in the pressure difference (i.e., towards zero) reduces waviness and the soft actuator surface becomes smoother. With positive pressure difference, a further increase in the pressure difference leads to additional shape waviness. There is no direct relation between the void diameter and the shape waviness.

**FIGURE 12:**
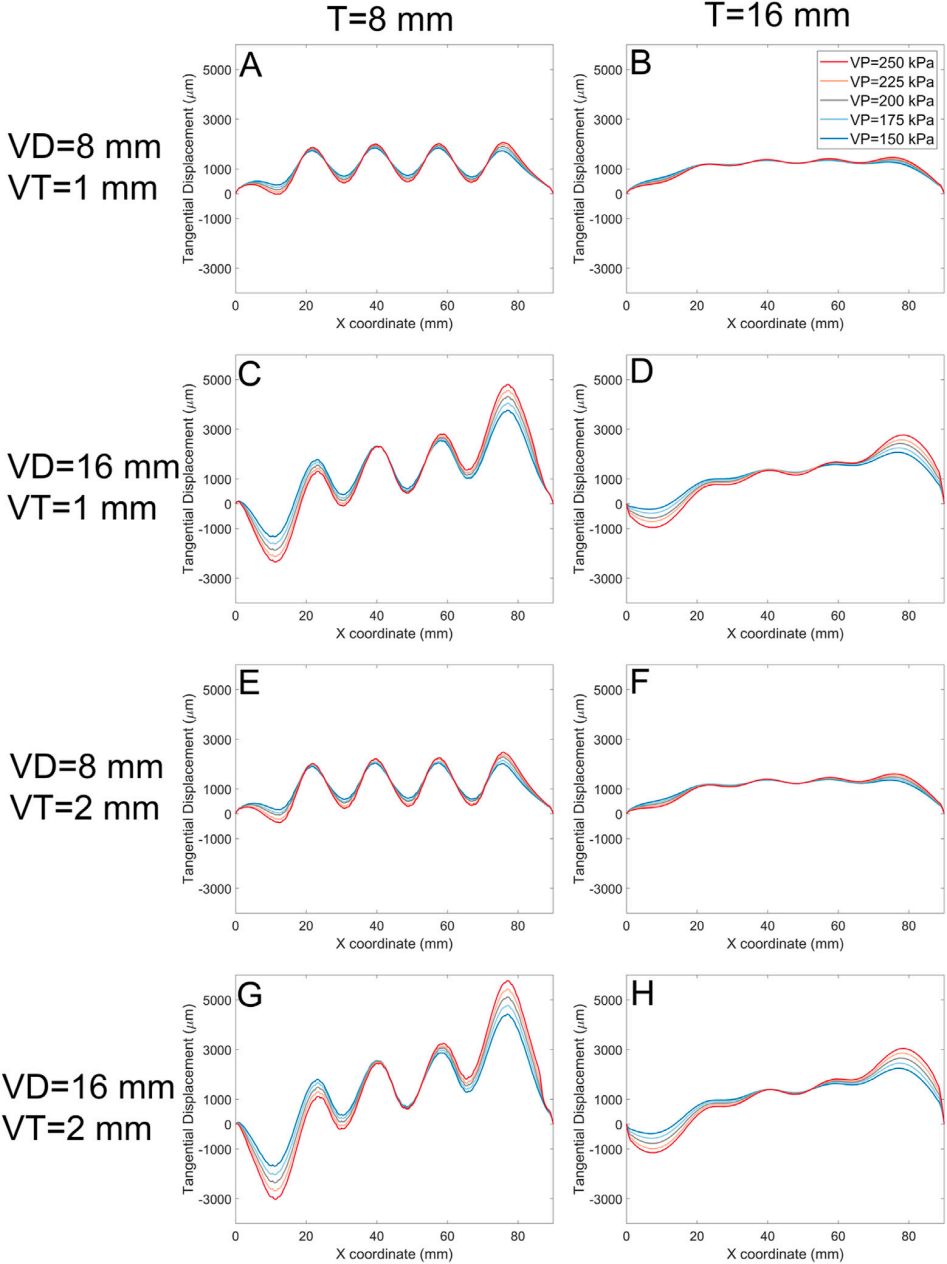
The tangential displacement (every node displacement along the shear stress direction) for models with 1 mm, and 2 mm void thickness (VT). Each panel represents data for a specific soft actuator thickness (T), void diameters (VD), and VT. Across panels, lines with the same colour represent the same soft actuator pressure. The first row of the panels **(A, B)** belongs to the data with the VD 8 mm, VT 1 mm, and T 8 mm, and 16 mm, respectively. The second row **(C, D)** demonstrates the results for the VD 16 mm, VT 1 mm, and T 8 mm, and 16 mm, respectively. The third row **(E, F)** demonstrates results for analysis with the VD 8 mm, VT 2 mm, and T 8 mm, and 16 mm, respectively. The fourth row **(G, H)** demonstrates FE results for the VD 16 mm, VT 2 mm, and T 8 mm, and 16 mm, respectively.

An important consideration is the performance of the actuator to shear loading. Figure 12 shows the result for the models when shear stress alone is applied to the surface of the soft actuators. The graphs in (Figure 12) depict the tangential displacement of each node along the *x*-axis (shear stress direction). The results show that Increasing the void diameter *VD* and/or void pressure V*P* results in increased shear displacement. However, increasing the soft actuator array thickness reduces the shear displacement magnitude. In some cases (Figures 12C, D, G, H) shear displacement is negative for some of the surface. By increasing the void thickness to 2 mm (Figures 12E, F, G, H), the general trend remains the same as the void thickness of 1 mm (Figures 12A–D). Nevertheless, the magnitude of shear displacement increased.

The maximum shear displacement for the soft actuator array with a void diameter of 8 mm of a thickness of 8 mm is about 2,500 µm (Figure 12E), but reaches about 6,000 µm when the void diameter becomes 16 mm (Figure 12G). For the void diameter of 8 mm (Figures 12E, F) the shear displacement at the centre of each void coordinate (i.e., *x* = 18 mm, *x* = 36 mm, *x* = 54 mm, and *x* = 72 mm) is almost the same. However, by increasing the void diameter more difference in the shear displacement at the void centres appears. For example (Figure 12G), shows that the magnitude maximum shear displacement for the last void centre is about 6,000 μm, while the magnitude of maximum shear displacement of the first void centre is about 1,200 µm. Increasing the soft actuator array thickness reduces the shear displacement difference between the centre of the first void and the last void.

**FIGURE 13:**
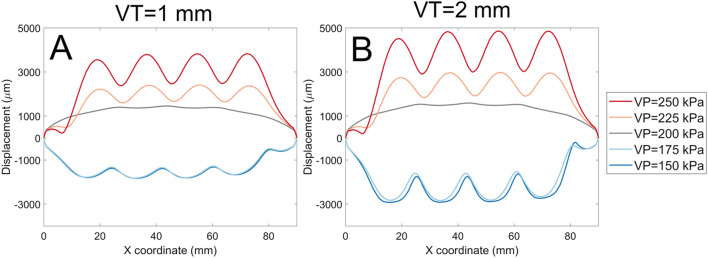
A comparison of surface displacement magnitude for the void thickness of 1 mm **(A)**, and void thickness of 2 mm **(B)** for the soft actuator array with the void diameter of 8 mm and thickness of 16 mm.

Often, external shear stress and normal stress will be applied simultaneously (Figure 13); depicts two examples of surface profile of the soft actuator array under a combination of shear and normal stress. The existence of shear stress shifts the surface profile to the right. The surface displacement for the void pressure of 200 kPa (gray line) is almost the same across all models. The main effect of increasing the void diameter is on the magnitude of surface displacement. For example, Figure 13 shows the surface displacement magnitude for soft actuator array with void thickness 1 mm (Figure 13A) and void thickness 2 mm (Figure 13B). The graph shows no evident difference in the surface shape, but the surface displacement increases by increasing the void thickness from 1 mm to 2 mm. For instance, for the void pressure of 250 kPa, the maximum surface displacement reached about 4,800 µm (Figure 13B) from about 3,200 µm (Figure 13A). The effect of void diameter and soft actuator thickness remained the same as discussed before on the surface shape of soft actuator array under combination of normal and shear stress.

**FIGURE 14:**
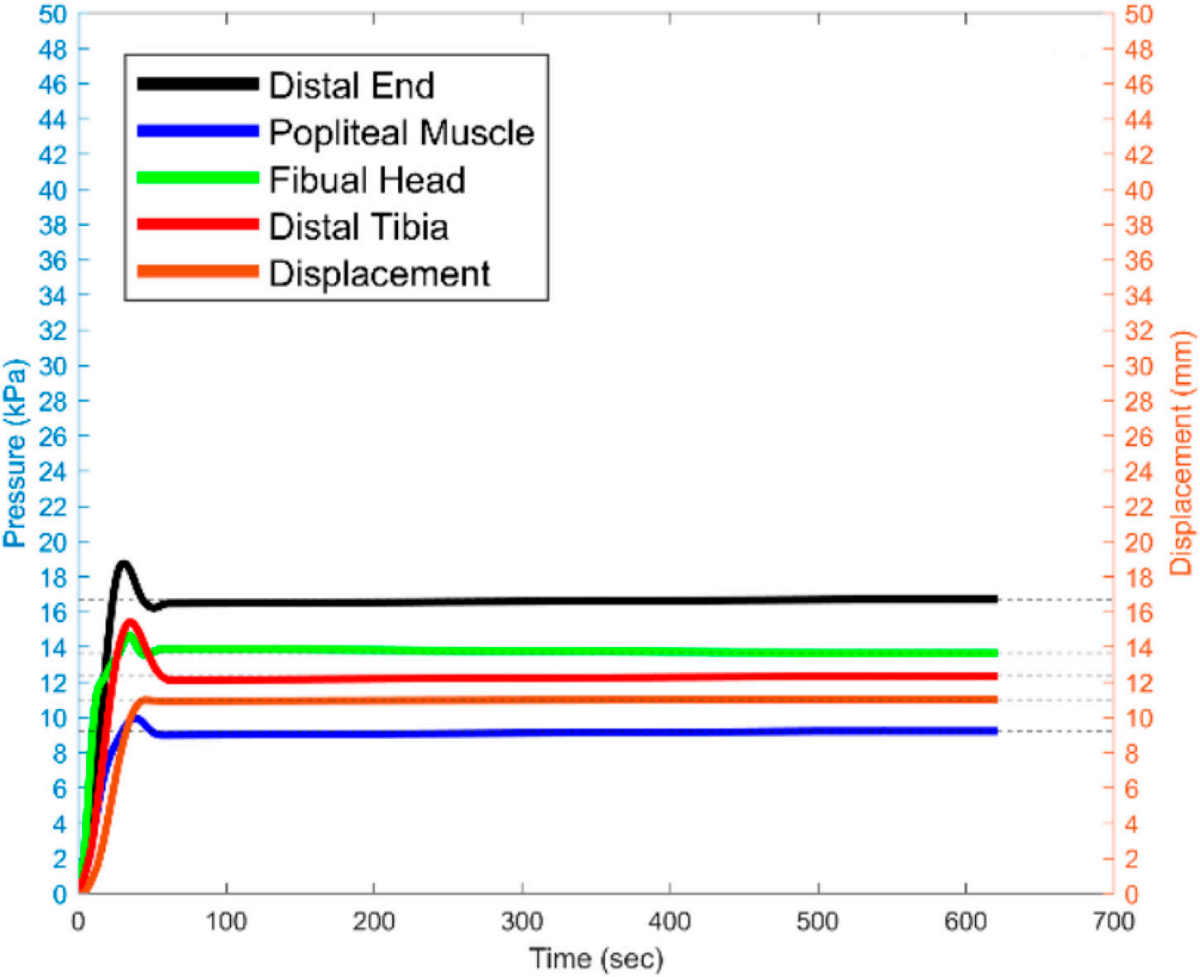
The recorded pressure for the sensitive areas, and voice coil actuator displacement for 600 s since the steady state moment the 15 N is being applied.

Figure 14 presents experimental data that characterises the pressure distribution across four critical regions of the residual limb: the distal end, popliteal muscle, fibular head, and distal tibia. It illustrates the recorded pressure profiles over a 600-s interval following the establishment of steady-state conditions, alongside the corresponding displacement of the voice coil actuator. The experiment details is discussed in (Mollaee et al., 2024).

The findings reveal that the distal end of the residual limb consistently experiences the highest pressure, particularly under a 15 N load applied vertically to the prosthetic socket. This elevated pressure concentration at the distal end indicates an imbalance in load distribution, which has significant implications for user comfort and may lead to tissue strain or injury with prolonged use. Such insights underscore the impact of socket design and load application on the comfort and functional mobility of amputees.

The results highlight the necessity for a pressure-optimised prosthetic socket design that minimises concentrated load, particularly in sensitive regions of the residual limb. Future iterations of socket design must prioritise the careful selection of materials and the optimisation of socket topology, particularly in the region of the distal end, to ensure a more uniform pressure distribution. This approach aims to enhance overall comfort, mitigate risks of pressure-induced tissue damage, and improve the quality of life for amputees by reducing discomfort during extended periods of use.

This study reports on a cost-efficient, easy manufacturing methodology for designing a dynamically reconfigurable socket, toward improving the socket’s comfort and fit for transtibial amputees by controlling the pressure distribution over the stump and compensating for residual limb volume fluctuation. Here, we predict the performance of an array of soft actuators, which can be used as a building block for creating a dynamically reconfigurable socket, or a similar deformable load-bearing surface. We have identified an appropriate hyperelastic constitutive model describing the soft actuator array and then explored the effect of different design parameters on the deformation of the array under realistic loading conditions.

In this study, we used a microrobot and 3D surface profiling to identify the Ogden N3 constitutive relation as the most accurate for predicting deformation of our pneumatic actuators (Table 3). We used the Ogden N3 hyperelastic constitutive model for our finite element models to investigate the effect of soft actuator array design parameters under loading conditions by simulating the actuator with loads developed at the prosthetic socket and residual limb interface. Maximum boundary normal and shear stress were applied to the surface of the soft actuator array based on values reported in the literature. Shear stress caused asymmetry in the surface shape. This was also observed in the FE model where the surface was squeezed and packed tightly at the end of the soft actuator surface where located at the endpoint of shear stress distribution (*x* = 90 mm), and manifested itself in the graphs steep negative slope (Figure 13).

Throughout the FE models, it could be seen that the soft actuator thickness had a significant effect on the surface shape and surface displacement under pressure. Increasing the soft actuator thickness resulted in a smoother surface (smaller shape waviness). Increasing the soft actuator thickness would also increase the soft actuator array bending stiffness (since it increases the second moment of inertia) and naturally requires greater pressure to deform, supporting the observation of an inverse relation between thickness and surface displacement.

The next design parameter studied was the void diameter. Increasing the void diameter increased the surface displacement magnitude (Figure 10). By increasing the void diameter, the same pressure would be applied to a bigger area, generating additional force and greater surface displacement. Despite this, increasing the void diameter does not essentially mean reducing the surface smoothness or shape waviness (Figure 11). Shape waviness change due to altering the void diameter significantly depend on the soft actuator thickness. Inflating the void would require the void to expand the layer on the top of it and lift the layer at the gap between the edge of the voids (Figure 2). Thus, there is a trade-off between the void diameter and soft actuator array thickness to change the shape waviness.

The input pressure for the soft actuator array has a strong influence on surface shape, and can be changed dynamically as a load on a socket varies. Increasing the pressure resulted in increased surface displacement and shape waviness. Due to the incompressibility assumption of the material, when the external normal stress was equal to the void pressure, the surface displacement was zero, and the soft actuator array was flat regardless of other design parameters. The surface deformation was positively correlated with pressure difference.

For the combination of design parameters that produced high shape waviness, the shear displacement has higher magnitudes. For example (Figure 12A), has a shape waviness of about 1 and displacement magnitude about 2000 µm. However, by decreasing the shape waviness to about 0.12 (Figure 12C), the displacement magnitude reduced to around 1,000 µm. The negative shear displacement shows that the tangential displacement as the result of the inflation was more than the displacement due to the shear stress, and always happens near the shear stress start point (*x* = 0 mm). The displacement magnitude for the Δ*P* = 0 kPa was almost the same since the shape waviness was approximately zero, and the only reason the surface was deformed was due to the existence of shear stress.

The shape waviness factor determines the resolution of the contact surface between the stump and the soft actuator array. By increasing the shape waviness, the resolution of the contact surface increases, which means that in case we need to locally add more pressure, for instance, redistribute the larger portion of the weight load to more tolerant stump areas, we can use the setting with higher shape waviness. The design setting resulting in smaller shape waviness (smoother surface) would enable the user to distribute the pressure over various regions of the stump uniformly.

It is desirable to minimise the socket’s weight and size and, to achieve this, it is preferable that the actuators would be thin. A very thin membrane thickness will also minimise the overall size. However, if the membrane is very thin, there will be very little resistance to forces tangential to the soft actuator and the socket may slip and increase the chance for skin irritation. Furthermore, if the void diameter is too small, it requires more input pressure to reach a certain displacement, and if it is too large, the resolution of pressure distribution at the socket/stump interface reduces. There is thus a tradeoff between the design parameters setting to achieve a certain soft actuator array surface deformation and shape. The dynamically reconfigurable socket includes several soft actuator arrays that, according to the amputee requirements, make it feasible to embed soft actuator arrays with different settings against different areas of the stump.

Our results show that a dynamically reconfigurable socket design can be optimised by using a combination of arrays of actuators. For the pressure tolerable areas of a residual limb, a soft actuator array with low thickness, such as 8 mm should be used to apply the load to pressure tolerable areas, such as the patellar tendon area. To provide sufficient control over applied load, the size of the void diameter should be big enough to cover the pressure tolerable area. A uniform pressure distribution may provide comfort and avoid tissue damage for the sensitive areas of the residual limb, such as the distal end of the tibia.

## Conclusion

This study demonstrated a practical design methodology investigation for a cost-efficient and easy manufacturing pneumatic soft actuator array, a building block for manufacturing a dynamically reconfigurable transtibial socket for controlling stump pressure distribution, and compensating for stump volume fluctuation, which are essential for improving the amputee’s quality of life. A mechanism for determining the hyperelastic properties of Ecoflex 0050 silicone was presented. This is a potential candidate for a soft actuator material given its reversible large strain capacity and softness. Deformation testing was performed with indentation and deformation caused by pressurising an internal pocket of air. To deal with the highly non-linear surface deformation, a new speckle imaging technique was used which was able to quantify the accuracy of the surface displacement to 40 µm. These measurements enable the Ogden N3 model to be identified as being able to predict actuator deformation to an accuracy of within 16%. This part of the study aimed to identify a suitable constitutive model to conduct FE analysis to identify appropriate soft actuator array configurations for use in a dynamically reconfigurable socket for lower limb amputees. This application determined some boundaries for interface stresses and pressures.

The FE model analysis showed that soft actuator array thickness would significantly affect the surface displacement magnitude and the surface shape. Void diameter and void thickness also play important roles in determining the surface displacement magnitude. However, their effect on the soft actuator array surface shape depends on its thickness. Input pressure was critical in determining the surface displacement and surface shape. It was observed that surface deformations are positively correlated with the pressure difference. Positive surface deformation enables us to control the surface profile and the pressure distribution over the stump by tuning it and reconfiguring the soft actuators’ surface shape. Soft actuators offer the prospect of a reconfigurable interface between a prosthetic socket and a limb stump of an amputee. This has the potential to improve prosthetic performance by improving comfort. However, the very nature of a soft actuator means that the interface will be flexible and to some extent compliant. This means that the deformation characteristics of the soft actuator will need to be determined accurately to provide the necessary support during active use. This paper presents the tools required to evaluate the properties of the soft actuators to allow their performance in this application to be evaluated with numerical models.

## Data Availability

The raw data supporting the conclusions of this article will be made available by the authors, without undue reservation.
